# PTTG1 expression is associated with hyperproliferative disease and poor prognosis in multiple myeloma

**DOI:** 10.1186/s13045-015-0209-2

**Published:** 2015-10-06

**Authors:** Jacqueline E. Noll, Kate Vandyke, Duncan R. Hewett, Krzysztof M. Mrozik, Rachel J. Bala, Sharon A. Williams, Chung H. Kok, Andrew CW Zannettino

**Affiliations:** Myeloma Research Laboratory, Department of Physiology, School of Medicine, Faculty of Health Sciences, University of Adelaide and Cancer Theme, South Australian Health and Medical Research Institute (SAHMRI), Adelaide, Australia; SA Pathology, Adelaide, Australia; Leukaemia Research Group, Cancer Theme, SAHMRI, Adelaide, Australia; Discipline of Physiology, School of Medicine, Faculty of Health Sciences, University of Adelaide, Cancer Theme, Level 5 South, SAHMRI, PO Box 11060, Adelaide, SA 5001 Australia

**Keywords:** PTTG1, Multiple myeloma, Proliferation, C57BL/KaLwRij

## Abstract

**Background:**

Multiple myeloma (MM) is an incurable haematological malignancy characterised by the clonal proliferation of malignant plasma cells within the bone marrow. We have previously identified pituitary tumour transforming gene 1 (*Pttg1*) as a gene that is significantly upregulated in the haematopoietic compartment of the myeloma-susceptible C57BL/KaLwRij mouse strain, when compared with the myeloma-resistant C57BL/6 mouse. Over-expression of PTTG1 has previously been associated with malignant progression and an enhanced proliferative capacity in solid tumours.

**Methods:**

In this study, we investigated PTTG1 gene and protein expression in MM plasma cells from newly diagnosed MM patients. Gene expression profiling was used to identify gene signatures associated with high PTTG1 expression in MM patients. Additionally, we investigated the effect of short hairpin ribonucleic acid (shRNA)-mediated PTTG1 knockdown on the proliferation of the murine myeloma plasma cell line 5TGM1 in vitro and in vivo.

**Results:**

*PTTG1* was found to be over-expressed in 36–70 % of MM patients, relative to normal controls, with high *PTTG1* expression being associated with poor patient outcomes (hazard ratio 2.49; 95 % CI 1.28 to 4.86; *p* = 0.0075; log-rank test). In addition, patients with high *PTTG1* expression exhibited increased expression of cell proliferation-associated genes including *CCNB1*, *CCNB2*, *CDK1*, *AURKA*, *BIRC5* and *DEPDC1*. Knockdown of *Pttg1* in 5TGM1 cells decreased cellular proliferation, without affecting cell cycle distribution or viability, and decreased expression of *Ccnb1*, *Birc5* and *Depdc1* in vitro*.* Notably, *Pttg1* knockdown significantly reduced MM tumour development in vivo, with an 83.2 % reduction in tumour burden at 4 weeks (*p* < 0.0001, two-way ANOVA)*.*

**Conclusions:**

This study supports a role for increased *PTTG1* expression in augmenting tumour development in a subset of MM patients.

**Electronic supplementary material:**

The online version of this article (doi:10.1186/s13045-015-0209-2) contains supplementary material, which is available to authorized users.

## Background

Multiple myeloma (MM) is characterised by the clonal proliferation of malignant plasma cells within the bone marrow (BM) and is the second most common haematological malignancy. The key clinical manifestations of MM include the development of painful osteolytic bone lesions, renal insufficiency, suppressed haematopoietic function and increased BM angiogenesis [1]. There are an array of clinical variants of the disease, ranging from the asymptomatic monoclonal gammopathy of undetermined significance (MGUS) and smouldering MM, to the more aggressive active MM and plasma cell leukaemia. Numerous genes, pathways and miRNAs have been identified in MM that function as predictive biomarkers of highly proliferative disease and likelihood of response to treatment [2–6]. Although the introduction of novel therapies has seen a significant improvement in the median survival of some groups of MM patients, the survival for some subgroups of patients, particularly those with highly proliferative disease, remains poor [7]. This highlights the need to identify new genes and pathways that may be involved in the pathophysiology of MM to aid in both prognosis and the development of novel therapeutics.

The C57BL/KaLwRij (KaLwRij) mouse strain, a closely related derivative of the C57BL/6 strain, is one of the best-studied pre-clinical animal models of MM. The KaLwRij strain is susceptible to developing benign monoclonal gammopathy and, in a small proportion of mice, MM at >2 years of age [8, 9]. Additionally, KaLwRij mice exhibit an inherent ability to support the growth of exogenous malignant plasma cells. The intravenous injection of murine myeloma cell lines, such as the KaLwRij-derived lines 5T33MM, 5T2MM and 5TGM1, into KaLwRij mice results in a myeloma-like disease that closely resembles human MM [10–14]. The mechanisms responsible for this susceptibility to the development of myeloma in this strain of mice remain largely unknown. Importantly, we [15, 16] and others [17] have previously utilised this model to identify key genes whose expression may play a role in the development of MM disease in these mice.

We have previously identified pituitary tumour transforming gene 1 (*Pttg1)* (also known as securin, EAP1 and TUTR1) as a gene that displays significantly increased expression in KaLwRij mice compared with C57BL/6 controls [15]. Notably, *PTTG1* is over-expressed in a vast array of malignancies including pituitary [18, 19], colorectal [20], thyroid [21] and lung [22] cancer, and high levels of PTTG1 are commonly associated with an enhanced proliferative capacity, increased tumour grade and high invasive potential [23]. PTTG1 is a key regulator of sister chromatid segregation during mitosis and, additionally, is involved in DNA damage repair [23]. An increase in *PTTG1* expression has previously been described in up to 63 % of MM patients [24, 25]; however, the role played by PTTG1 in MM disease development has not been determined. In the present study, we confirm up-regulation of *PTTG1* in MM plasma cells from a subset of MM patients compared with both MGUS and healthy controls and show that elevated *PTTG1* expression is associated with an increase in cell cycle-related gene expression and is associated with poor survival. Furthermore, knockdown of *Pttg1* decreases cellular proliferation in vitro and reduces myeloma tumour burden in vivo in the KaLwRij model of MM. Collectively, these data support a role for PTTG1 in promoting MM disease pathogenesis, likely through cell cycle- and proliferation-related pathways.

## Results

### *PTTG1* is over-expressed in the C57BL/KaLwRij mouse model of myeloma

**Fig. 1:**
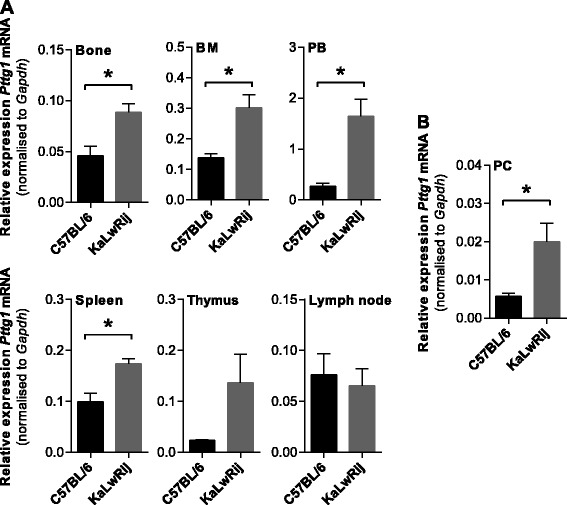
*Pttg1* expression is increased in haematopoietic tissues of C57BL/KaLwRij mice. **a** Bone, bone marrow (BM), peripheral blood (PB), spleen and thymus and combined popliteal, inguinal and axillary lymph node tissues were isolated from C57BL/6 and C57BL/KaLwRij mice (*n* = 3/group) and total RNA extracted. *Pttg1* mRNA expression was determined by qRT-PCR analysis. *p < 0.05, *t* test. **b** CD138^+^ plasma cells (PC) were isolated from the BM of C57BL/6 and C57BL/KaLwRij mice (*n* = 3/group). *Pttg1* mRNA was significantly increased in KaLwRij-derived plasma cells compared with C57BL/6 controls. Graphs depict mean + SEM; *p < 0.05, *t* test

In order to identify genes that may play a role in the development of myeloma, we previously compared the transcriptome of the bone/BM of KaLwRij mice to that of the genetically related C57BL/6 strain using microarray [15]. Using this approach, we identified *Pttg1* as a gene with significantly increased expression (2.9-fold; *p* = 0.00037, LIMMA) within the bone/BM of KaLwRij mice compared with C57BL/6 controls [15]. Quantitative real-time PCR (qRT-PCR) was subsequently used to assess the relative messenger ribonucleic acid (mRNA) expression levels of *Pttg1* in a range of tissues derived from the C57BL/6 and KaLwRij mice (*n* = 3/group). As seen in Fig. 1a, *Pttg1* mRNA levels were significantly increased in the bone, BM, peripheral blood and spleen of KaLwRij mice, when compared with those of C57BL/6 controls (*p* < 0.05, *t* test). Although an increase in *Pttg1* expression was also noted in the thymus, this did not reach significance (*p* = 0.11, *t* test). Furthermore, there was a significant increase in *Pttg1* expression in CD138^+^ plasma cells derived from KaLwRij mice compared with those from C57BL/6 controls (Fig. 1b; *p* = 0.045, *t* test). Together, these data confirm that *Pttg1* expression levels are up-regulated in the bone, haematopoietic tissues and plasma cells of KaLwRij mice.

### *PTTG1* is over-expressed in MM patients and is associated with poor survival

**Fig. 2:**
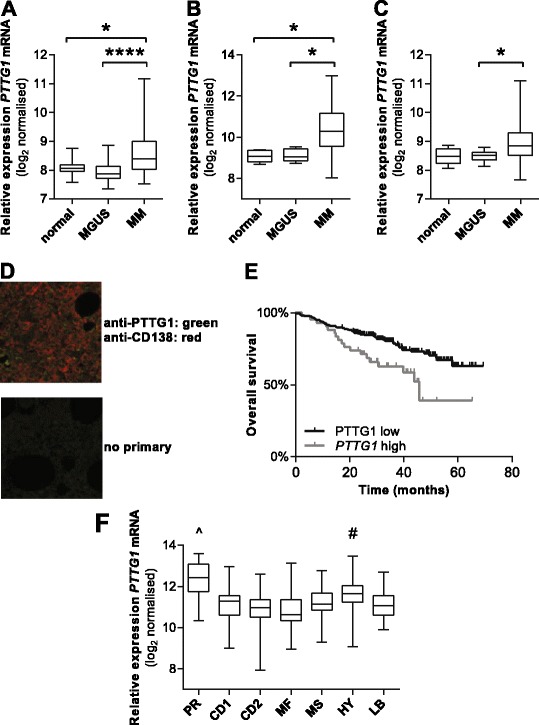
*PTTG1* is over-expressed in MM patients. In silico analysis was performed on publically available gene expression datasets from CD138^+^ plasma cells isolated by MACS from MM (*n* = 73) and MGUS (*n* = 22) patients and healthy controls (*n* = 15) (E-GEOD-6477) (**a**), MM (*n* = 155) and MGUS (*n* = 5) patients and healthy controls (*n* = 5) (E-MTAB-363) (**b**) and MM (*n* = 133) and MGUS (*n* = 11) patients and healthy controls (*n* = 5) (E-GEOD-16122) (**c**). Box and whiskers plots show the median and interquartile ranges for each cohort. *p < 0.05; **** *p* < 0.0001; Kruskal-Wallis test with Dunn’s multiple comparison tests. **d** Representative image of a BM trephine section from an MM patient stained with anti-CD138 (*red*) and anti-PTTG1 (*green*), showing plasma cell-specific protein expression of PTTG1. A negative (*no primary antibody*) control is shown. **e** Kaplan-Meier plot of *PTTG1* high patients (quartile 4; *n* = 71) vs *PTTG1* low patients (quartiles 1–3; *n* = 214) (TT2 patients from GSE4581). **f** MM patients from GSE4581 (*n* = 414) were stratified into subgroups based on the UAMS criteria; namely, patients characterised by increased proliferation-related genes (PR), chromosomal translocations involving cyclin D1 and cyclin D3 (CD1 and CD2), MAF (MF) or MMSET (MS), as well as patients exhibiting hyperdiploidy (HY) and decreased prevalence of lytic bone disease (LB) [4]. The expression of *PTTG1* was analysed in each subset. Box and whiskers plots show the median and interquartile ranges for each cohort; ^*p* < 0.0001 relative to CD1, CD2, MF, MS, HY and LB; #*p* < 0.01 relative to CD2, MF, MS, LB; Kruskal-Wallis test with Dunn’s multiple comparison tests

To examine whether MM patients display increased *PTTG1* expression, we performed in silico analyses in three independent publically available microarray datasets (E-GEOD-6477; E-GEOD-16122; E-MTAB-363) comparing the gene expression profiles of CD138^+^-isolated plasma cells from newly diagnosed MGUS and MM patients and normal controls. In E-GEOD-6477 and E-MTAB-363, *PTTG1* expression was significantly increased in the MM patient cohort compared with the normal controls (*p* < 0.05) and MGUS patients (*p* < 0.05; Fig. 2a, b). In E-GEOD-16122, *PTTG1* expression was significantly increased in MM patients compared with MGUS patients (*p* < 0.05; Fig. 2c). Approximately 38–70 % (38.4 % [28/73], E-GEOD-6477; 70.3 % [109/155], E-MTAB-363; 68.4 % [66/133], E-GEOD-16122) of MM patients expressed *PTTG1* at levels higher than the normal range (mean + 2SD of the normal cohort expression). We subsequently isolated CD138^+^ plasma cells from diagnostic MM patient BM (*n* = 11) using CD138-MACS, total RNA was isolated and *PTTG1* mRNA expression examined by qRT-PCR. Four of the 11 patients (36.4 %) were found to express *PTTG1* in the purified plasma cells (Additional file 1: Figure S1). Using dual-colour immunohistochemistry, we also confirmed PTTG1 protein expression within CD138^+^ plasma cells in BM trephines from two *PTTG1*-expressing MM patients (Fig. 2d). Consistent with previous reports [24, 26], PTTG1 protein was predominantly cytoplasmic.

Collectively, these data suggest that over-expression of *PTTG1* is a feature of MM disease in approximately 36–70 % of MM patients. In order to determine whether the increased expression of *PTTG1* in MM patients was related to patient survival, newly diagnosed MM patients enrolled in the total therapy 2 (TT2) trial, from publically available microarray dataset GSE4581, were stratified based on *PTTG1* expression levels. The quartile with the highest *PTTG1* expression was classified as *PTTG1* high (*n* = 71 patients), while the remaining patients (*n* = 214) were classified as *PTTG1* low. Subsequent analysis of overall survival identified a significantly poorer survival in the *PTTG1* high group (hazard ratio 2.49; 95 % CI 1.28 to 4.86; *p* = 0.0075; log-rank [Mantel-Cox] test; Fig. 2e).

When MM patients in dataset GSE4581 were partitioned into gene expression profiling-defined subgroups (UAMS classifications) [4], elevated *PTTG1* was found to be associated with specific disease subtypes (Fig. 2f). Specifically, *PTTG1* expression was elevated in the PR subgroup, characterised by expression of proliferation-related genes, when compared with the subgroups characterised by chromosomal translocations involving cyclin D1 and D3 (CD1 and CD2), MAF (MF) or MMSET (MS), by hyperdiploidy (HY) or by a decreased prevalence of lytic bone disease (LB) (Fig. 2f). Additionally, significantly elevated *PTTG1* was observed in the HY group, when compared with the CD2, LB, MF and MS subgroups.

### *PTTG1* expression in MM patients is associated with an increase in expression of cell cycle-associated genes

**Fig. 3:**
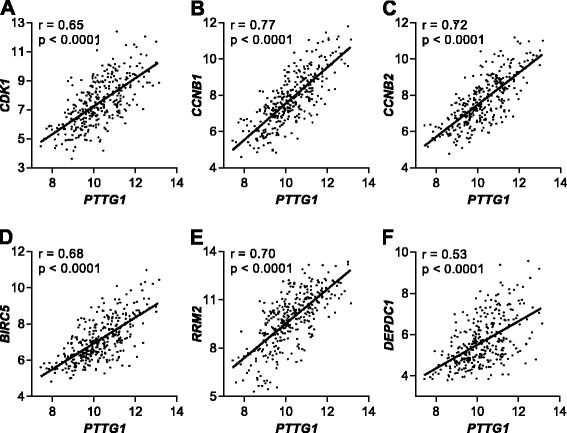
*PTTG1* expression strongly correlates with expression of cell proliferation-related genes in MM patients. *PTTG1* expression in 328 newly diagnosed MM patients (E-GEOD-19784) plotted against expression of cell cycle-related genes *CDK1* (**a**), *CCNB1* (**b**), *CCNB2* (**c**), *BIRC5* (**d**), *RRM2* (**e**) and the non-cell cycle gene *DEPCD1* (**f**). *r* and *p* values are shown for Pearson correlation analyses

As elevated *PTTG1* expression was shown to be associated with poor outcomes in MM patients, we next compared MM plasma cell gene expression between *PTTG1* high and *PTTG1* low MM patients in four independent microarray datasets. Twenty-nine genes were found to be significantly down-regulated, and 1459 genes significantly up-regulated (excluding *PTTG1*) in at least one dataset (Fisher’s *p* value <0.05). Of these, 155 genes were significantly up-regulated, and no genes were down-regulated, in all four datasets. Of the 119 of these genes that were classified in DAVID, there was an enrichment for cell cycle-related genes, with 82/119 (68.9 %) being genes associated with mitosis. Genes up-regulated by more than twofold, all with a strong (*p* < 0.0001) positive correlation (Spearman) with *PTTG1* expression, included key cell cycle regulators (eg. *CCNB1*, *CCNB2*, *CDK1*, *CKS2*), genes associated with DNA replication (eg. *MCM2*, *GINS1*, *RRM2*), response to DNA damage (eg. *CHEK1*, *PBK*), mitotic spindle and microtubule organisation (eg. *AURKA*, *NEK1*, *PRC1*), chromosome segregation during mitosis (eg. *CENPA*, *CENPH*, *PENPK*, *BIRC5*) and ubiquitin ligase activity and protein catabolism (eg. *UBE2C*, *CDC20*, *MAD2L1*, *DTL*) (Table 1; Fig. 3). Other non-cell cycle genes associated with high *PTTG1* include *DEPDC1* and the histone demethylase *EZH2*.

**Table 1:** Gene significantly upregulated more than twofold in *PTTG1* high MM patients

Probeset ID^a^	Gene symbol	Fisher’s *p* value^b^	Fold change^c^ [mean (95 % CI)]
*Regulation Of cell cycle*			
213226_at	*CCNA2*	2.20 × 10^−31^	2.32 (1.84–2.80)
214710_s_at	*CCNB1*	2.18 × 10^−40^	4.07 (2.61–5.53)
202705_at	*CCNB2*	1.98 × 10^−41^	3.54 (1.93–5.14)
203213_at	*CDK1*	9.11 × 10^−37^	3.91 (2.44–5.39)
1555758_a_at	*CDKN3*	1.63 × 10^−40^	2.90 (1.68–4.13)
204170_s_at	*CKS2*	2.02 × 10^−35^	2.88 (2.69–3.07)
218350_s_at	*GMNN*	1.03 × 10^−32^	2.32 (1.71–2.93)
204825_at	*MELK*	5.20 × 10^−35^	2.67 (1.88–3.47)
*DNA replication*			
206102_at	*GINS1*	6.74 × 10^−29^	2.99 (1.91–4.07)
202107_s_at	*MCM2*	1.04 × 10^−33^	2.33 (1.79–2.87)
201930_at	*MCM6*	5.41 × 10^−20^	2.03 (1.32–2.74)
201202_at	*PCNA*	1.74 × 10^−22^	2.36 (1.81–2.91)
204127_at	*RFC3*	3.82 × 10^−17^	2.12 (1.27–2.97)
209773_s_at	*RRM2*	2.80 × 10^−36^	4.15 (2.38–5.92)
1554696_s_at	*TYMS*	7.15 × 10^−41^	2.47 (0.95–4.00)
*Response to DNA damage*			
205394_at	*CHEK1*	1.33 × 10^−36^	2.74 (1.88–3.61)
213007_at	*FANCI*	2.04 × 10^−24^	2.25 (1.04–3.47)
202503_s_at	*KIAA0101*	1.34 × 10^−38^	3.59 (2.23–4.96)
223700_at	*MND1*	9.45 × 10^−41^	2.03 (1.44–2.92)
219148_at	*PBK*	3.18 × 10^−38^	3.62 (1.60–5.64)
205909_at	*POLE2*	5.33 × 10^−25^	2.08 (1.49–2.67)
204146_at	*RAD51AP1*	2.74 × 10^−26^	2.41 (1.56–3.26)
219258_at	*TIPIN*	2.66 × 10^−33^	2.06 (1.70–2.41)
204033_at	*TRIP13*	4.71 × 10^−38^	2.45 (1.87–3.04)
*Mitotic spindle and microtubule organisation*			
208079_s_at	*AURKA*	4.49 × 10^−35^	2.74 (2.22–3.26)
204162_at	*NDC80*	1.96 × 10^−32^	3.23 (1.85–4.62)
204641_at	*NEK2*	1.24 × 10^−29^	2.84 (1.65–4.03)
218039_at	*NUSAP1*	9.20 × 10^−37^	3.47 (1.29–5.66)
218009_s_at	*PRC1*	4.37 × 10^−31^	2.54 (1.69–3.39)
222077_s_at	*RACGAP1*	5.24 × 10^−31^	3.01 (1.90–4.13)
209891_at	*SPC25*	3.04 × 10^−40^	2.84 (2.62–3.06)
200783_s_at	*STMN1*	1.27 × 10^−34^	2.33 (1.73–2.94)
204822_at	*TTK*	1.30 × 10^−34^	2.66 (1.03–4.28)
204026_s_at	*ZWINT*	4.49 × 10^−29^	3.09 (1.01–5.17)
*Sister chromatid segregation*			
202095_s_at	*BIRC5*	8.63 × 10^−38^	2.60 (1.94–3.26)
204962_s_at	*CENPA*	7.33 × 10^−38^	2.97 (2.14–3.81)
231772_x_at	*CENPH*	2.98 × 10^−32^	2.12 (1.76–2.47)
222848_at	*CENPK*	5.40 × 10^−31^	2.90 (1.86–3.94)
219555_s_at	*CENPN*	1.53 × 10^−33^	2.06 (1.45–2.67)
226936_at	*CENPW*	2.43 × 10^−39^	2.40 (1.39–3.41)
218663_at	*NCAPG*	2.21 × 10^−34^	2.61 (1.44–3.79)
213599_at	*OIP5*	6.22 × 10^−41^	2.99 (2.39–3.59)
*Ubiquitin ligase activity*			
202870_s_at	*CDC20*	1.02 × 10^−36^	2.43 (2.28–2.58)
222680_s_at	*DTL*	4.43 × 10^−29^	2.88 (1.22–4.55)
203362_s_at	*MAD2L1*	1.01 × 10^−32^	3.07 (1.92–4.23)
202954_at	*UBE2C*	2.48 × 10^−37^	2.20 (1.15–3.24)
223229_at	*UBE2T*	4.11 × 10^−30^	2.30 (0.82–3.78)
*Chromatin modification*			
203358_s_at	*EZH2*	1.21 × 10^−24^	2.41 (1.63–3.20)
227212_s_at	*PHF19*	1.18 × 10^−29^	2.38 (1.59–3.17)
*Other*			
222958_s_at	*DEPDC1*	2.47 × 10^−25^	2.32 (1.31–3.32)
226980_at	*DEPDC1B*	2.52 × 10^−33^	2.37 (2.00–2.74)
225834_at	*FAM72A/FAM72B/FAM72C/FAM72D*	1.10 × 10^−29^	3.31 (1.01–5.62)
228069_at	*MTFR2*	1.36 × 10^−31^	2.07 (1.62–2.53)
235113_at	*PPIL5*	3.17 × 10^−27^	2.08 (1.53–2.63)
229551_x_at	*ZNF367*	1.30 × 10^−20^	2.20 (1.48–2.93)

^a^Genes are shown for which statistically significant differences were observed in all of the four datasets analysed. Data is shown for the Affymetrix probeset with the lowest *p* value for each gene

^b^Fisher’s method was used to combine the *p* values across the four datasets

^c^Mean fold change observed for *PTTG1* high vs *PTTG1* low patients across the four datasets

### *Pttg1* knockdown in 5TGM1 cells inhibits cell proliferation in vitro

**Fig. 4:**
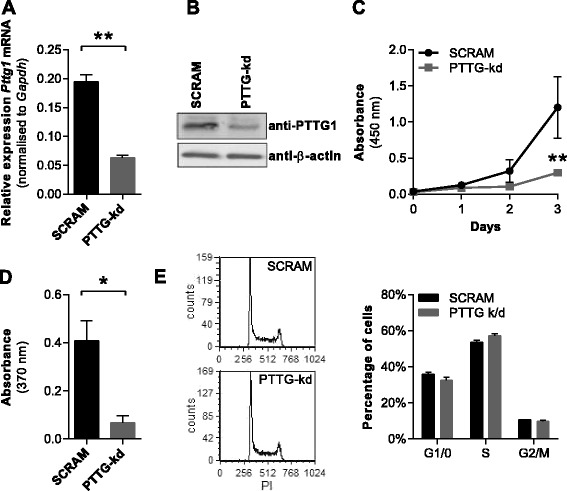
Pttg1 knockdown reduces 5TGM1 proliferation in vitro. **a** A 70 % knockdown of *Pttg1* mRNA was confirmed in 5TGM1-PTTG-kd cells compared with 5TGM1-SCRAM controls by qRT-PCR. **b** Reduced protein expression of Pttg1 was confirmed by Western blot. **c** Cells were seeded at 1 × 10^5^ cells/mL in a 96-well plate, and cell numbers over 3 days were quantitated by WST-1 assay, read at 450 nm. **d** Cells were seeded at 4 × 10^5^ cells/mL in a 96-well plate, BrdU substrate was added and BrdU incorporation was quantitated after 2 h by ELISA, measured by absorbance at 370 nm. *p < 0.05, *t* test. **e** Cells were seeded at 4 × 10^5^ cells/mL in a six-well plate and cultured for 24 h, and cell cycle distribution was assessed following PI staining. Representative FACS plots (PI histograms) are shown. Graphs depict mean + SEM of three independent experiments

Consistent with the elevated expression of *Pttg1* in KaLwRij-derived plasma cells and the KaLwRij mouse tumour origin of the 5TGM1 cell line [27, 28], 5TGM1 cells express high levels of *Pttg1* (data not shown). Using lentiviral transduction, we stably introduced a short hairpin ribonucleic acid (shRNA) within an mCherry-tagged vector to specifically knockdown *Pttg1* in luciferase-expressing 5TGM1 cells. *Pttg1* expression levels, as assessed by qRT-PCR (Fig. 4a) and Western blot (Fig. 4b), were reduced by 70 % in the knockdown cell line (denoted 5TGM1-PTTG-kd) when compared with a scrambled shRNA control line (5TGM1-SCRAM). These cell lines were used for subsequent in vivo and in vitro experiments.

As high *PTTG1* expression in MM patients correlates with increased expression of cell cycle- and proliferation-associated genes, we next examined whether *Pttg1* may play a role in modulating cell cycle progression and proliferation in murine myeloma plasma cells. After 3 days of culture, cell number, as determined by water-soluble tetrazolium salt (WST-1) assay, was significantly decreased by 75 % in the 5TGM1-PTTG-kd cells when compared with the 5TGM1-SCRAM controls (Fig. 4c, *p* < 0.01, two-way ANOVA with Sidak’s multiple comparison tests). Furthermore, the proliferative capacity of the 5TGM1-PTTG-kd line in vitro was decreased by 83 % compared with the 5TGM1-SCRAM control, as determined by bromodeoxyuridine (BrdU) incorporation over 2 h (Fig. 4d, *p* = 0.018, *t* test). However, there was no effect of *Pttg1* knockdown on cell cycle distribution, as determined by propidium iodide (PI) staining (Fig. 4e, *p* = 0.998, two-way ANOVA). Furthermore, cell viability, as assessed by trypan blue exclusion, was not affected by *Pttg1* knockdown (data not shown). Taken together, these results suggest that elevated expression of *Pttg1* in the malignant 5TGM1 cells may be associated with increased cell proliferation.

### Pttg1 knockdown reduces the expression of proliferation-related genes in the 5TGM1 mouse myeloma cell line

**Fig. 5:**
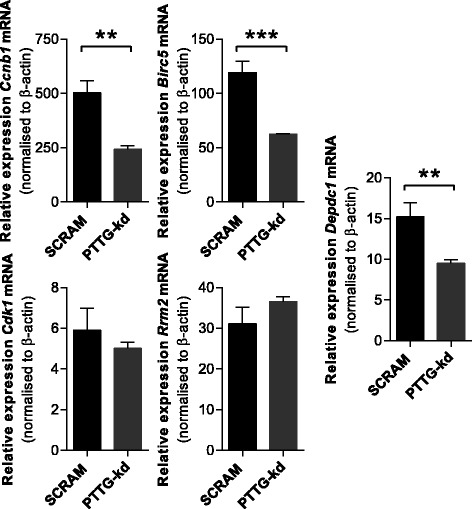
Pttg1 knockdown results in deregulation of proliferation-related genes in 5TGM1 cells. Expression of *Ccnb1*, *Birc5*, *Cdk1*, *Rrm2* and *Depcd1* was quantitated in 5TGM1-PTTG-kd cells compared with 5TGM1-SCRAM controls by qRT-PCR. Graphs depict mean + SD of triplicates from a single experiment. *p < 0.05, *t* test

In order to elucidate a potential mechanism through which loss of Pttg1 inhibits proliferation, we examined the expression of a selection of proliferation-related genes identified in the human patient datasets (see Table 1) in the 5TGM1-PTTG1-kd cell line compared with the 5TGM1-SCRAM control. Our findings show that expression of genes encoding the cell cycle regulator *Ccnb1* (*p* = 0.0039, *t* test) and the kinetochore-associated protein *Birc5* (*p* = 0.0008, *t* test) were decreased by 52 and 48 %, respectively, in the PTTG1-kd cells, compared with the scramble control cells (Fig. 5). In contrast, expression of genes encoding the cell cycle regulator *Cdk1* and the deoxyribonucleotide synthesis enzyme *Rrm2* were not affected by *Pttg1* knockdown (*p* = 0.2391, *t* test). Additionally, expression of *Depdc1*, which has previously been implicated in MM [29], was decreased by 38 % (*p* = 0.0055, *t* test) in the PTTG1-kd cells.

### *Pttg1* knockdown in 5TGM1 cells reduces tumour burden in vivo

**Fig 6:**
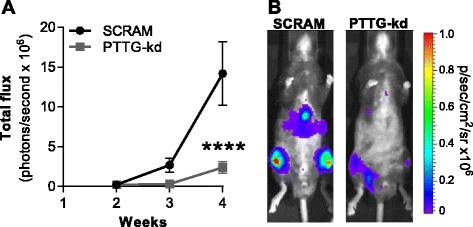
Pttg1 knockdown reduces tumour growth in vivo. **a** Total tumour burden was measured at 2-, 3- and 4-weeks post-tumour cell inoculation using bioluminescence imaging techniques. A significant reduction in total tumour burden was observed in the Pttg kd group (*n* = 15) compared with SCRAM controls (*n* = 10); ****p < 0.001, two-way ANOVA with Sidak’s post-test. **b** Representative bioluminescent images of mice injected with 5TGM1-SCRAM control (*left*) and 5TGM1-PTTG-kd (*right*) cells at 4-weeks post-tumour cell inoculation are shown

To ascertain whether the observed decrease in proliferation in the 5TGM1-PTTG-kd cells corresponded with reduced tumour growth in vivo, the 5TGM1-PTTG-kd and 5TGM1-SCRAM cell lines were injected i.v. into C57BL/KaLwRij mice and tumour burden was monitored at weekly intervals by bioluminescent imaging. As seen in Fig. 6, tumour burden was decreased by 83 % in the *Pttg1* knockdown group compared with the control group at 4 weeks (*p* < 0.0001, two-way ANOVA with Sidak’s post-test), suggesting that high basal expression of *Pttg1* in 5TGM1 cells is important for in vivo tumour growth.

## Discussion

Over-expression of PTTG1 has previously been linked to neoplastic transformation in a wide range of cell types [23, 30–32]. Increased expression of *PTTG1* has been extensively studied in pituitary adenomas, as well as a range of other endocrine cancers (reviewed by [33]). By comparison, little is known about the role PTTG1 may play in haematological malignancies. Early studies demonstrated that *PTTG1* was highly expressed in approximately 70 % of patients with leukaemia, lymphoma or other myelodysplastic diseases but not in healthy donors [26, 34]. More recently, high PTTG1 protein expression has been observed in 63 % of MM patients [24, 25]; however, the biological and prognostic significance of PTTG1 over-expression was not investigated in these studies. In the current study, we show a significant increase in *PTTG1* expression in human MM patients, with approximately 36–70 % of patients showing increased expression of *PTTG1* above that seen in healthy controls, findings which were subsequently confirmed by analysis of our own MM patient specimens. Importantly, increased *PTTG1* expression was associated with poor overall survival, suggesting that high *PTTG1* expression may be implicated in MM disease severity and poor patient outcome. Additionally, our analyses show that PTTG1 is upregulated in MM patients, but not in the asymptomatic precursor MGUS, suggesting an association with disease progression.

Chromosomal rearrangements and duplications are early events in the development of myeloma with the presence of genomic instability being associated with poor prognosis [35]. In this study, elevated *PTTG1* was specifically observed in a subgroup of MM patients displaying a hyperdiploid phenotype (HY group). This is consistent with the role of PTTG1 in regulating sister chromatid separation during mitosis [36, 37] as dysregulation of this key function commonly leads to genomic instability [38–42]. Over-expression of PTTG1 promotes dysregulated chromosome segregation resulting in aneuploidy in human cancer cell lines [40, 41, 43]. The chromosomal instability induced by PTTG1 over-expression has been proposed as a mechanism whereby PTTG1 drives malignant transformation [44]. Interestingly, gene expression profiling studies have shown that the presence of chromosomal instability in newly diagnosed myeloma patients is associated with a gene expression profile that includes upregulation of *PTTG1* [35]. These data suggest that expression of *PTTG1* in MM patients may contribute to the development of chromosome duplications characteristic of the HY group.

In addition to its association with hyperdiploidy, we found a significant increase in *PTTG1* expression in subset of patients with a proliferation-related gene expression profile (PR group), which is associated with high-risk disease and poor prognosis [4]. Elevated *PTTG1* has previously been identified as part of a gene signature that is associated with increased proliferative index and is an independent predictor of poor prognosis in newly diagnosed MM patients [45]. The increase in *PTTG1* in MM patients with highly proliferative disease is consistent with data from other systems which show that increased expression of *PTTG1* correlates with high levels of cellular proliferation [46–48]. While, in the short term, over-expression of PTTG1 prevents exit from mitosis, leading to cell cycle arrest and increased cell death [49, 50], sustained; stable over-expression of PTTG1 generally leads to enhanced cellular proliferation [32, 51, 52]. Notably, our *Pttg1* knockdown studies in the KaLwRij-derived 5TGM1 myeloma plasma cell line resulted in a reduction in cellular proliferation in vitro, as well as decreased tumour development in vivo*.* This is consistent with a number of animal knockout and knockdown studies in a range of different cell types, which show that reduction of *PTTG1* expression inhibits cell proliferation [52–59]. Taken together, these data are consistent with increased expression of *Pttg1* being a core requirement for the growth of malignant plasma cells in some patients.

In support of the pro-proliferative role of PTTG1 in myeloma, analysis of four independent MM patient gene expression datasets revealed that the majority (68.9 %) of genes significantly up-regulated in *PTTG1* high patients had proliferation-related functions, specifically cell cycle regulation, DNA replication, mitotic spindle formation, chromosome segregation and DNA damage pathways. These included 20 of the top 50 genes originally identified as being upregulated in the PR subgroup [4]. Notable exclusions include the cancer/testis antigens *MAGEA1*, *MAGEA3*, *MAGEA6*, *GAGE1*, *GAGE2*, *GAGE4* and *GAGE5*, which were originally identified as being strongly upregulated in the PR subgroup [4] but are not upregulated in our *PTTG1* high patients. This, combined with the strength of the association between *PTTG1* expression and the expression of cell cycle-associated genes, suggests that the upregulation of proliferation-related genes with high *PTTG1* expression is not simply due to an increased representation of PR patients in this group.

Indeed, we found that knockdown of *Pttg1* was associated with downregulation of cell cycle regulatory genes in the 5TGM1 cell line, suggesting a potential mechanism for the decreased proliferation observed in these cells. Cell cycle genes *CcnB1* (cyclin B1) and *Birc5* (survivin), which were among the most highly up-regulated genes identified in our patient analysis, were down-regulated by approximately 50 % by *Pttg1* knockdown in the 5TGM1 cells. Both *CCNB1* and *BIRC5* have been identified as part of gene expression signatures predictive of high-risk disease and poor prognosis in MM patients in several studies [4, 5, 45, 60–63]. *PTTG1*, *CCNB1* and *BIRC5* expression are under tight transcriptional control during cell cycle progression, being switched on during G_2_/M phase [46, 49, 64, 65]. While this suggests that downregulation of *Ccnb1* and *Birc5* expression following *Pttg1* could be a consequence of a decrease in G_2_/M phase cells, we saw no change in cell cycle distribution in the PTTG1-kd cells to support this. Cyclin B1, an essential regulator of cell cycle transition during mitosis, has previously been identified as a *PTTG1*-regulated gene whose up-regulation is associated with enhanced proliferation in keratinocytes [52]. BIRC5 is an inhibitor of apoptosis proteins (IAP) family member which regulates microtubule dynamics and chromosomal segregation during mitosis. Over-expression and knockdown studies in human myeloma cell lines have implicated BIRC5 in proliferation and protection of MM cells from apoptosis [62, 64, 66]. Taken together, these data suggest a potential role for cyclin B1 and BIRC5 in PTTG1-mediated tumour growth.

In addition to its role as a securin during chromatid segregation, PTTG1 can directly regulate gene expression. PTTG1 has been shown to physically interact with p53, repressing its transcriptional activity [36]. Additionally, p53 expression can be transcriptionally and translationally repressed by transient over-expression of PTTG1 [67]. Notably, some of the genes found to strongly correlate with *PTTG1* expression in this study, including *CDK1*, *CCNB1*, *CCNB2* and *BIRC5* are known to be inhibited downstream of p53 [68–70]; p53-mediated regulation of gene expression down stream of PTTG1 could be a focus of future studies. However, we found no correlation between PTTG1 and p53 expression in MM patients in any of the datasets examined (data not shown), suggesting that PTTG1 is unlikely to transcriptionally downregulate p53 expression in plasma cells from MM patients. Additionally, our data suggests that *PTTG1* upregulation in MM patients is not a consequence of deletion of the p53 locus 17p13, or associated *TP53* mutations [71], which is observed in approximately 10 % of patients and is itself associated with poor prognosis [72–74].

In addition to cell cycle genes, we found that expression of *DEPDC1* (Dishevelled, EGL-10, Pleckstrin domain containing 1) was significantly up-regulated in *PTTG1* high patients. Importantly, *Pttg1* knockdown in myeloma cells leads to a 40 % reduction in *DEPDC1* expression. While the function of DEPDC1 is unknown, its expression has been associated with poor prognosis in lung cancer [75] and advanced disease in breast cancer [76]. Knockdown of DEPDC1 or inhibition using a specific peptide results in decreased cell proliferation and apoptosis in bladder cancer cell lines [77, 78]. In myeloma, *DEPDC1* expression has been associated with poor prognosis [29]. In addition, shRNA-mediated *DEPCD1* knockdown in human myeloma cell lines was shown to significantly inhibit cell proliferation and induce accumulation in G2/M in *TP53* wild-type cells and marked apoptosis in *TP53* mutant cells [29]. These data suggest that regulation of *DEPDC1* expression may be a mechanism whereby PTTG1 regulates cell proliferation in myeloma.

Although studies have identified a role for PTTG1 in regulating epithelial-mesenchymal transition (EMT) [79–81] and recent studies by Azab and colleagues [82] have identified a role for the EMT processes in the dissemination and homing of MM plasma cells to the BM, we showed no association between the expression of EMT-related genes and high PTTG1 expression in MM patients. In addition, PTTG1 has previously been shown to directly mediate pro-angiogenic pathways through regulation of secreted factors VEGF and FGF-2 [19, 83, 84], which in turn are commonly expressed in MM and associated with increased disease severity [85–87]. However, our analyses did not identify an increase in these, or other, secreted pro-angiogenic factors in the presence of increased PTTG1 expression in MM patients. These data suggest that PTTG1 is unlikely to function through EMT or angiogenic pathways in modulating MM disease.

## Conclusions

In summary, we have identified *PTTG1* as a gene which is over-expressed in the MM-susceptible KalwRij mouse and in MM patients with hyperdiploidy or with hyperproliferative disease, suggesting a role in MM disease development. Knockdown of *Pttg1* significantly inhibited the proliferation of myeloma cells in vitro, with an associated decrease in the expression of mitosis-related genes, and slowed tumour development in vivo. While expression of *PTTG1* has previously been noted in gene expression signatures defining myeloma patients with highly proliferative disease [4] and chromosomal instability [35] and, by association, poor outcomes, this is the first study to show that *PTTG1* expression alone is sufficient to identify a subset of patients with poor overall survival. Collectively, our data suggest that the poor prognosis associated with *PTTG1* expression is due to a hyperproliferative state in these patients, which may result from the PTTG1-mediated upregulation of key drivers of cell cycle progression.

## Materials and methods

### Mouse tissue and plasma cell isolation

C57BL/6 mice were obtained from the Animal Resources Centre (Perth, Australia). C57BL/KaLwRij mice, originally kindly provided by Prof. Andrew Spencer (Monash University, Melbourne, Australia), were bred and housed at the SA Pathology Animal Care Facility (Adelaide, Australia). Tissues from C57BL/6 and C57BL/KaLwRij mice were snap frozen in liquid nitrogen and homogenised in TRIzol (Life Technologies, Carlsbad, CA). Blood was obtained from mice by cardiac puncture, collected in microfuge tubes containing 50 μL 0.5 M EDTA and centrifuged for 10 min at 500*g* and the cell pellet resuspended in TRIzol by vigorous vortexing. Femora and tibiae from age- and sex-matched C57BL/6 and C57BL/KaLwRij mice were extracted and cleaned thoroughly. A 21-gauge needle was inserted into the BM cavity, and the BM was flushed with ice-cold PFE (PBS/2 %FCS/2 mM EDTA). The resulting cell suspension was subjected to Ficoll density gradient separation and collected in 10 mL PFE, followed by centrifugation at 300*g* for 5 min at 4 °C. BM cells were immediately lysed in TRIzol, or CD138^+^ plasma cells were isolated by FACS. Briefly, cells were resuspended at 1 × 10^7^ cells/mL in PFE and blocked with 110 μg/mL murine gamma globulin (Jackson Laboratories, Bar Harbor, ME) for 30 min at 4 °C. Cells were stained for 30 min at 4 °C protected from light with rat anti-mouse CD138 (R & D Systems, Minneapolis, MN) or an isotype control, washed twice with PFE and stained with secondary goat anti-rat IgG PE (Southern Biotech, Birmingham, AL) for 30 min at 4 °C protected from light. Cells were washed three times with PFE, followed by sorting for CD138^+^ cells on a FACSAria II (BD Biosciences, San Jose, CA). Total RNA was isolated from sorted cells using an RNAqueous Micro kit (Life Technologies).

### Microarray analysis

For comparison of *PTTG1* expression in CD138+ BM plasma cells, isolated by CD138-MACS, from newly diagnosed MM or MGUS patients or normal controls, three independent datasets were used: E-GEOD-6477 (normal, *n* = 5; MGUS, *n* = 11; MM, *n* = 133 [61]), E-GEOD-16122 (normal, *n* = 15; MGUS, *n* = 22; MM, *n* = 73 [88]) and E-MTAB-363 (normal, *n* = 5; MGUS, *n* = 5; MM, *n* = 156 [89]). Analysis of *PTTG1* expression in different gene expression-defined (UAMS) patient subsets was conducted in GSE4581 (*n* = 414) [4]. Analysis of patient survival in *PTTG1* high and *PTTG1* low newly diagnosed MM patients was carried out using GSE4581 analysing patients included in the total therapy 2 (TT2) trial (*n* = 256). Four independent datasets were used for analysis of gene expression in PTTG1 high (quartile 4) and PTTG1 low (quartiles 1–3) patient subsets in CD138-selected BM plasma cells from newly diagnosed MM patients: E-GEOD-19784 (*n* = 328) [90], E-GEOD-26863 (*n* = 304) [91], E-MTAB-317 (*n* = 226) [45] and E-MTAB-363 (*n* = 156). E-MTAB-363, E-GEOD-26863, E-MTAB-317 and GSE4581 were conducted on Affymetrix GeneChip Human Genome U133 plus 2.0 arrays; E-GEOD-6477 and E-GEOD-16122 were conducted on Affymetrix GeneChip Human Genome U133A arrays. For all datasets except GSE4581, raw microarray data (CEL files) were downloaded from ArrayExpress (EMBL-EBI) and were normalised by RMA using the bioconductor package (affy) [92] and R (version 3.03) and log_2_ transformed. One patient in E-MTAB-363 (V0681) failed quality control (normalised unscaled standard error [NUSE] >1.05) and was excluded, and the remaining 165 files were re-normalised. For GSE4581, MAS5-normalised data were downloaded from the Gene Expression Omnibus (GEO) and were log_2_ normalised prior to analysis. GO annotation (http://www.geneontology.org) and DAVID [93, 94] were used to classify genes by related function.

### MM patient samples

BM trephines and aspirates were collected, with informed consent, from patients with MM at the time of diagnosis and prior to initiation of therapy. This study was approved by the Royal Adelaide Hospital Human Research Ethics Committee (application # RAH 030206 and 131133).

### CD138^+^ plasma cell isolation from MM patients

CD138^+^ plasma cells were isolated from diagnostic MM patient BM samples using CD138 microbeads (Miltenyi Biotech, Auburn, CA) as previously described [15]. Briefly, cryopreserved human BM samples were thawed into 10 mL DMEM (high glucose) with 15 % FCS and DNase I (80 U/mL). Samples were centrifuged at 300*g* for 10 min and supernatant removed. The cell pellet was resuspended in MACS buffer (2 mM EDTA, 0.5 % deionised BSA in PBS) and CD138 microbeads added, followed by incubation on ice for 15 min. Cell-bead conjugates were washed in 1 mL MACS buffer and centrifuged at 300*g* for 10 min. Cells were resuspended in MACS buffer, applied to a pre-rinsed MS column and washed three times with MACS buffer and eluted in 1 mL. Subsequent FACS analysis confirmed >85 % CD138^+^ following MACS. Total RNA was isolated using an All Prep DNA/RNA Micro Kit (Qiagen, Valencia, CA).

### Dual colour staining BM trephines

Paraffin-embedded BM trephine sections were mounted on silicane-coated slides and dried. Endogenous peroxidase was blocked with 0.5 % H_2_O_2_ in methanol at room temperature for 30 min, followed by blocking with 3 % normal horse serum (NHS) for 30 min. Slides were incubated with anti-PTTG1 antibody (diluted 1:50; DCS-280; Abcam) at room temperature overnight. Slides were washed twice in PBS and incubated with biotinylated anti-mouse IgG (Vector Laboratories, Burlingame, CA) diluted 1:250 for 30 min at room temperature, washed in PBS and incubated with streptavidin Alexa Fluor 488 (diluted 1:500; Life Technologies) diluted 1:500 for 1 h at room temperature. Slides were re-blocked with 3 % NHS for 30 min and incubated with mouse anti-human CD138 (diluted 1:40; MI15, Dako, Denmark) overnight. Slides were washed twice with PBS followed by incubation for 1 h at room temperature with anti-mouse Alexa Fluor 594 (diluted 1:500, Life Technologies) and mounted in aqueous mounting solution. Images were taken on a Zeiss LSM 700 confocal system (Zeiss, Oberkocken, Germany) at ×40 magnification.

### Cell lines

The murine myeloma plasma cell line 5TGM1 was kindly provided by Ass. Prof. Claire Edwards (University of Oxford, Oxford, UK), and was maintained in Iscove’s modified Dulbecco’s medium (Sigma) with 20 % FCS. Unless otherwise specified, all culture medium was supplemented with 2 mM l-glutamine, 100 U/mL penicillin, 100 μg/mL streptomycin, 10 mM HEPES buffer (Life Technologies) and 1 mM sodium pyruvate. 

### Generation of stable PTTG1 knockdown lines

To generate stable knockdown cell lines, an RNA duplex targeting murine *Pttg1* (GGGAAATTGCAGGTTTCAACG) was cloned into the pFIV-H1-mCherry vector. A scrambled sequence was used as a control. pFIV-H1-mCherry was created by excising the *GFP* cassette from pFIV-H1-GFP (System Biosciences, Mountain View, CA) using XbaI and SalI and replacing it with the *mCherry* cassette from pMSCV-mCherry. Following lentiviral infection of 5TGM1-luc cells (expressing a dual GFP and luciferase reporter construct [16, 96, 97]), single-cell clones were generated from the top 10 % GFP- and mCherry-expressing cells using preparative cell sorting and the automatic cell deposition unit on a Beckman Coulter Epics Altra HyperSort, using Expo MultiComp Software version 1.2B (Beckman Coulter, Miami, FL). Clonal 5TGM1-PTTG-kd and 5TGM1-SCRAM lines were used for subsequent in vitro and in vivo assays.

### Real-time PCR

Total RNA was isolated using TRIzol (Life Technologies) as per the standard protocol (unless otherwise specified). For mouse and human CD138^+^ plasma cells, RNA was reverse transcribed using Sensiscript (Qiagen). For all other tissues and cell lines, RNA (1 μg) was reverse transcribed with Superscript III (Life Technologies) as per the manufacturer’s protocol. Real-time PCR was conducted on the Corbett Rotorgene using the following primers: human β-actin (F: 5'-TTGCTGACAGGATGCAGAAG-3' and R: 5'-AAGGGTGTAAAACGCAGCTC-3'), human *PTTG1* (F: 5'-CGGCCTCAGATGAATGCGGCT-3' and R: 5'-TTGATTGAAGGTCCAGACCCCAGC-3'), mouse *Gapdh* (F: 5'- ACCCAGAAGACTGTGGATGG-3' and R: 5'-CAGTGAGCTTCCCGTTCAG-3'), mouse β-actin (F: 5'-TTG CTGACAGGATGCAGAAG-3' and R: 5'-CAGTGAGCTTCCCGTTCA-3'), mouse *Pttg1* (F: 5'-GCTCCTGATGATGCCTACCC-3' and R: 5'-CGCCATTCAAGGGGAGAAGT-3'), mouse *Ccnb1* (F: 5'-GATGATGGGGCTGACCCAAA-3' and R: 5'-ACATGGTCTCCTGAAGCAGC-3'), mouse *Cdk1* (F: 5'-GTCCGTCGTAACCTGTTGAG-3' and R: 5'-TGACTATATTTGGATGTCGAAG-3') [98], mouse *Rrm2* (F: 5'-GATTTAGCCAAGAAGTTCAAGTTACAG-3' and R: 5'-TCACACAAGGCATAGTTTCAATAGC-3') [99], mouse *Birc5* (F: 5'-GAACCCGATGACAACCCGAT–3' and R: 5'-TGGTCTCCTTTGCAATTTTGTTCT-3') and mouse *Depcd1* (F: 5'-AGCTGCAGTGGAGAAACATCT-3' and R: 5'-TGGTCTCCTTTGCAATTTTGTTCT-3'). Gene expression was represented relative to β-actin or *Gapdh* expression, calculated using the 2^-ΔCT^ method.

### Western blot analysis

Cells were lysed in lysis buffer (1 % NP-40, 20 mM HEPES, 150 mM NaCl, 10 % glycerol, 2 mM Na_3_VO_4_, 10 mM Na_4_P_2_O_7_, 2 mM NaF and Complete EDTA-free Protease Inhibitor Cocktail (Roche, Mannheim, Germany)). Total lysate (10 μg) was loaded on an 11 % acrylamide gel and subjected to SDS-PAGE. Proteins were transferred to PVDF membrane overnight. Membrane was incubated for 1 h in blocking buffer (Tris-buffered saline containing 0.1 % Tween20 and 2.5 % ECL Blocking Agent (GE Healthcare, Little Chalfont, UK)) and for 2 h at RT with mouse monoclonal anti-PTTG1 antibody (DCS-280; Abcam; Cambridge, MA) diluted 1:1000 in blocking buffer, followed by alkaline phosphate-conjugated anti-mouse IgG (Millipore, Billerica, MA) diluted 1:4000 in blocking buffer for 1 h at RT. Proteins were visualised using ECL detection reagent (GE Healthcare) on a Typhoon FLA 7000 IP^2^ (GE Healthcare).

### Proliferation assays

For WST-1 assays, 5TGM1 cells (PTTG-kd or SCRAM controls) were seeded at 1 × 10^5^ cells/well in triplicate in 96-well plates and were incubated at 37 °C with 5 % CO_2_. At 24-h intervals, WST-1 reagent (Roche) was added to the cells and incubated for 2 h prior to reading absorbance at 450 nm.

For BrdU assays, 5TGM1 cells were seeded at 4 × 10^5^ cells/well in triplicate in a 96-well plate. BrdU (Roche) was immediately added to the cells and incubated for 2 h at 37 °C with 5 % CO_2_. BrdU incorporation was measured using a BrdU Cell Proliferation ELISA kit (Roche) as per manufacturer’s protocol and absorbance measured at 370 nm.

For cell cycle analysis, cells were seeded at 4 × 10^5^ cells/well in a six-well plate and incubated for 24 h at 37 °C with 5 % CO_2_. Cells were fixed in ice-cold 70 % (*v*/*v*) ethanol, washed twice in PBS and stained with propidium iodide (PI; 40 μg/mL; Sigma) containing 20 μg/mL RNase A (Qiagen) for 30 min prior to analysis on a Gallios flow cytometer (Beckman Coulter). Cell cycle distribution was analysed using FCS Express version 4.

### Animals

Ethical approval for this study was obtained from the SA Pathology/Adelaide Health Service Animal Ethics Committee (application # 136/10). C57BL/KaLwRij mice at 6–8 weeks of age were injected with 5 × 10^5^ luciferase-expressing 5TGM1-luc cells (5TGM1-SCRAM or 5TGM1-PTTG-kd) in 100-μl sterile PBS via the tail vein. At weekly intervals, mice were administered luciferin (150 mg/kg) i.p. and imaged using the Xenogen IVIS 100 bioluminescence imaging system (Caliper Life Sciences, Hopkinton, MA) until termination of the experiment at day 28, prior to the development of lethal disease (hind limb paralysis) [16, 96]. Total tumour burden was measured as total flux (photons/second) for each animal using Living Image software (PerkinElmer, Waltham, MA), as described previously [15, 16, 97, 100–103].

### Statistical analyses

Statistical analysis was performed using GraphPad Prism version 6.03 for Windows (GraphPad Software, San Diego, CA). Variance between patient groups was assessed using Kruskal-Wallis tests with Dunn’s multiple comparison tests. In each of the E-GEOD-19784, E-GEOD-26863, E-MTAB-317 and E-MTAB-363 datasets, gene expression was compared in *PTTG1* high and *PTTG1* low patients, using *t* tests with Bonferroni’s correction for multiple testing and *p* values from the four datasets were combined using Fisher’s method. Survival curves were compared using the log-rank (Mantel-Cox) test with hazard ratios calculated using the Mantel-Haenszel calculation. In vivo data, WST-1 assays and cell cycle distribution were analysed by two-way ANOVA with Sidak’s multiple comparison tests. qRT-PCR data and BrdU incorporation were compared between groups using unpaired two-tailed *t* tests. A *p* value of 0.05 was considered statistically significant. Unless otherwise described, all plots depict mean + SEM of three independent experiments.

## Additional file

Additional file 1: Figure S1. PTTG1 expression is upregulated in MM PC. *PTTG1* expression was quantitated in CD138-selected BM PC from newly diagnosed MM patients (*n* = 11) using qRT-PCR. Graph shows mean + SD of triplicates from a single experiment.
