# Supplementary figures and images for: PTTG1 expression is associated with hyperproliferative disease and poor prognosis in multiple myeloma
Source: J Hematol Oncol. 2015 Oct 6;8:106. doi: 10.1186/s13045-015-0209-2 (PMC4595141; doi:10.1186/s13045-015-0209-2)

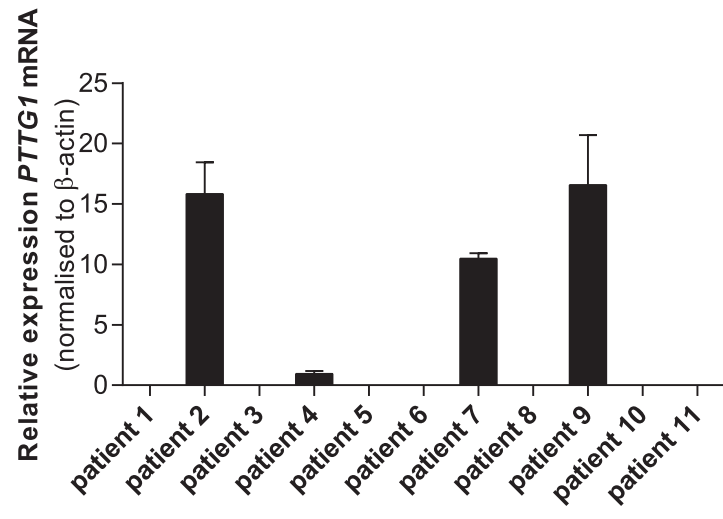

Supplement: Additional file 1: Figure S1. — PTTG1 expression is upregulated in MM PC. PTTG1 expression was quantitated in CD138-selected BM PC from newly diagnosed MM patients (n = 11) using qRT-PCR. Graph shows mean + SD of triplicates from a single experiment. [file 13045_2015_209_MOESM1_ESM.pdf]
